# Supplementary material for: Evolutionarily recent transcription factors partake in human cell cycle regulation
Source: Cell Genom. 2025 Jun 23;5(8):100923. doi: 10.1016/j.xgen.2025.100923 (PMC12366659; doi:10.1016/j.xgen.2025.100923)
Supplement: Document S1. Figures S1–S6 [file mmc1.pdf]

Cell Genomics, Volume *5*

Supplemental information

Evolutionarily recent transcription factors partake in human cell cycle regulation

Cyril Pulver, Romain Forey, Alex R. Lederer, Martina Begnis, Olga Rosspopoff, Joana Carlevaro-Fita, Filipe Martins, Evarist Planet, Julien Duc, Charlène Raclot, Sandra Offner, Alexandre Coudray, Arianna Dorschel, and Didier Trono

Supplemental Figures and Legends

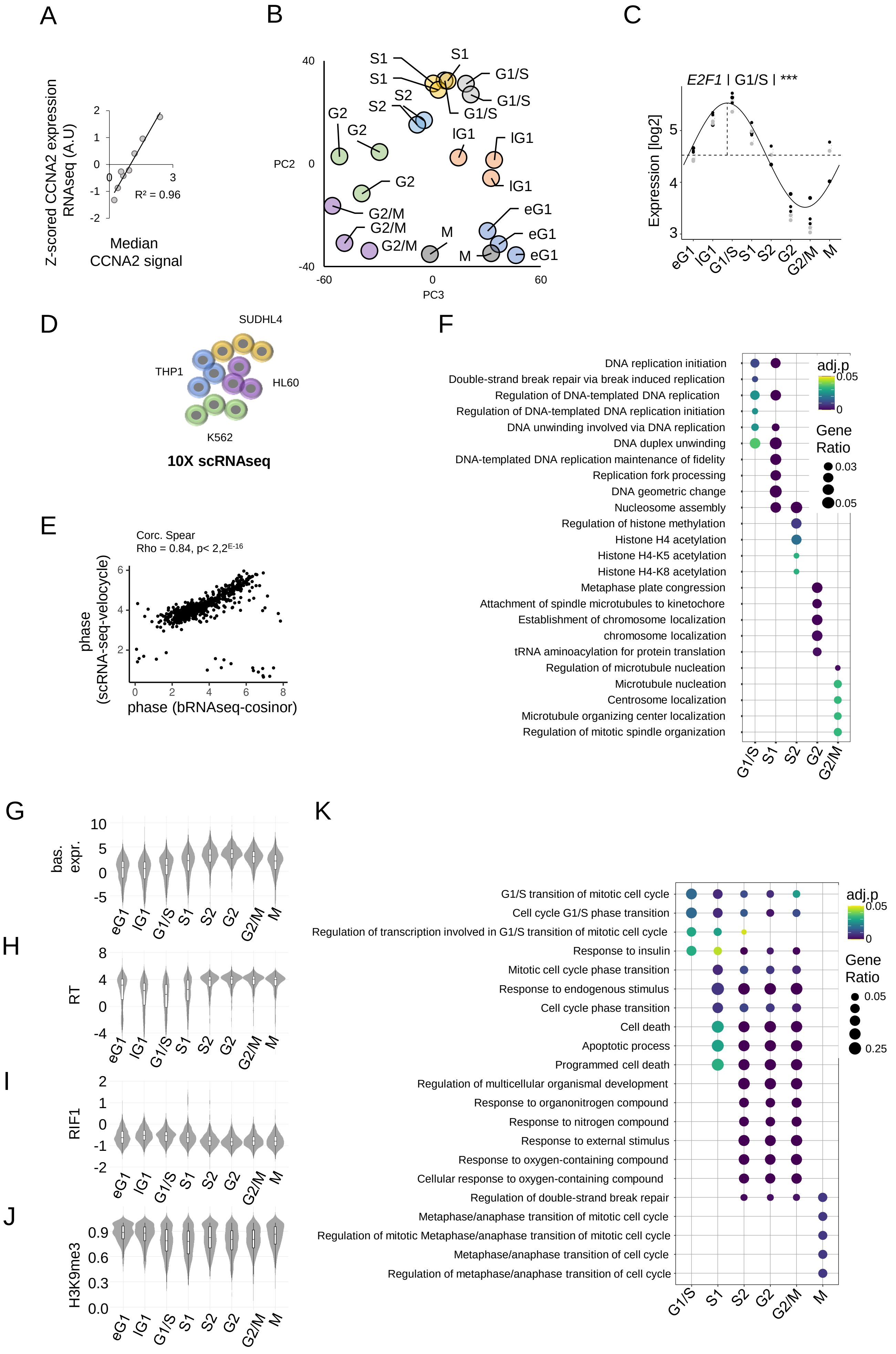
A
Z-scored CCNA2 expression RNAseq (A.U)
Median CCNA2 signal
R² = 0.96
B
PC2
PC3
S1
S2
G1/S
IG1
G2
G2/M
M
eG1
C
E2F1 | G1/S | ***
Expression [log2]
eG1
IG1
G1/S
S1
S2
G2
G2/M
M
D
SUDHL4
THP1
HL60
K562
10X scRNAseq
E
Corc. Spear
Rho = 0.84, p< 2,2E-16
phase (scRNA-seq-velocycle)
phase (bRNAseq-cosinor)
F
DNA replication initiation
Double-strand break repair via break induced replication
Regulation of DNA-templated DNA replication
Regulation of DNA-templated DNA replication initiation
DNA unwinding involved via DNA replication
DNA duplex unwinding
DNA-templated DNA replication maintenance of fidelity
Replication fork processing
DNA geometric change
Nucleosome assembly
Regulation of histone methylation
Histone H4 acetylation
Histone H4-K5 acetylation
Histone H4-K8 acetylation
Metaphase plate congression
Attachment of spindle microtubules to kinetochore
Establishment of chromosome localization
chromosome localization
tRNA aminoacylation for protein translation
Regulation of microtubule nucleation
Microtubule nucleation
Centrosome localization
Microtubule organizing center localization
Regulation of mitotic spindle organization
adj.p
Gene Ratio
G
bas. expr.
H
RT
I
RIF1
J
H3K9me3
K
G1/S transition of mitotic cell cycle
Cell cycle G1/S phase transition
Regulation of transcription involved in G1/S transition of mitotic cell cycle
Response to insulin
Mitotic cell cycle phase transition
Response to endogenous stimulus
Cell cycle phase transition
Cell death
Apoptotic process
Programmed cell death
Regulation of multicellular organismal development
Response to organonitrogen compound
Response to nitrogen compound
Response to external stimulus
Response to oxygen-containing compound
Cellular response to oxygen-containing compound
Regulation of double-strand break repair
Metaphase/anaphase transition of mitotic cell cycle
Regulation of mitotic Metaphase/anaphase transition of mitotic cell cycle
Metaphase/anaphase transition of cell cycle
Regulation of metaphase/anaphase transition of cell cycle
adj.p
Gene Ratio

Figure S1. Synchronisation- and transgene-free cell cycle chronogram of gene expression, related to Figure 1

(A) Correlation between CCNA2 expression (RNA-seq) and CCNA2 signal (FACS) across the eight gates defined Fig. 1A.
(B) Principal Component Analysis (PCA) on RNA-seq data (batch-corrected normalized counts) performed on the eight cell fractions defined in Fig. 1A. Replicates, corresponding with cell cycle fractions, are grouped by colours.
(C) E2F1 expression in K562. Rhythmicity was estimated by fitting E2F1 logged norm. counts to a sinusoid[1], with experimental batch as a covariate. Batch-corrected expression values are shown as pairs of grey (raw) and black (corrected) dots connected by a dotted curved segment. Horizontal dotted line: baseline expression. Vertical dotted line: acrophase. A rounded phase of peak expression is indicated on top, with the adj. p-value of the corresponding rhythmicity F-test. "***" indicates adj. $p < 0.005$.
(D) scRNA-seq performed on an untreated mix of four cell lines[2]. Cell cycle phases were estimated using *VeloCycle*[3].
(E) Circularized Spearman's rank correlation between phases of peak expression estimated from the bulk RNA-seq chronogram of cell cycle gene expression and the scRNA-seq in (C) using *VeloCycle*. Note that axes are circular, i.e. 0 is synonymous with 8 on the x-axis.
(F) Enrichment of rhythmic genes across Gene Ontology (GO) Biological Process (BP) terms. Dot areas represent the proportion of rhythmic genes peaking in each phase found across the enriched GO terms, and colour scale the Benjamini-Hochberg adjusted p-values (hypergeometric test).
(G) Baseline expression of rhythmic genes across phases.
(H) RT index of rhythmic genes across phases.
(I) RIF1 binding of rhythmic genes across phases.
(J) H3K9me3 promoter coverage of rhythmic genes across phases.
(K) Enrichment of phase-enriched DBPs across GOBP terms

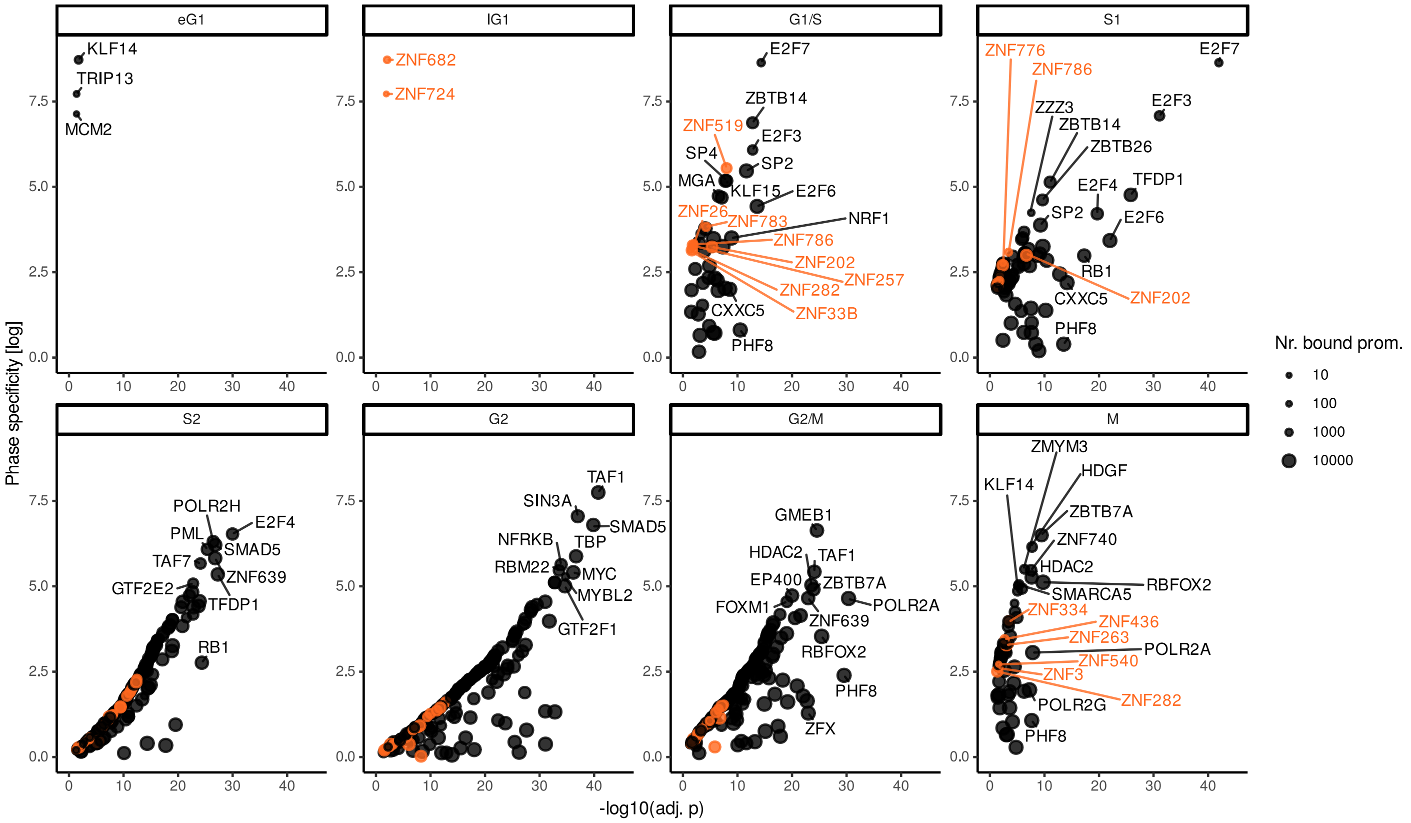

eG1
lG1
G1/S
S1
S2
G2
G2/M
M
KLF14
TRIP13
MCM2
ZNF682
ZNF724
E2F7
ZBTB14
ZNF519
E2F3
SP4
SP2
MGA
KLF15
E2F6
ZNF26
ZNF783
NRF1
ZNF786
ZNF202
ZNF257
ZNF282
CXXC5
ZNF33B
PHF8
ZNF776
ZZZ3
ZBTB26
E2F4
TFDP1
RB1
POLR2H
PML
SMAD5
TAF7
ZNF639
GTF2E2
TAF1
SIN3A
NFRKB
TBP
RBM22
MYC
MYBL2
GTF2F1
GMEB1
HDAC2
EP400
ZBTB7A
FOXM1
POLR2A
RBFOX2
ZFX
ZMYM3
HDGF
ZNF740
SMARCA5
ZNF334
ZNF436
ZNF263
ZNF540
ZNF3
POLR2G
Phase specificity [log]
-log10(adj. p)
Nr. bound prom.
10
100
1000
10000

Figure S2. Select KZFPs are enriched at phase-specific promoters, related to Figure 1

Phase-specific enrichment of DBPs across rhythmic promoters. Dot areas represent the number of genes whose promoters are bound by the DBP. KZFPs are highlighted in orange.

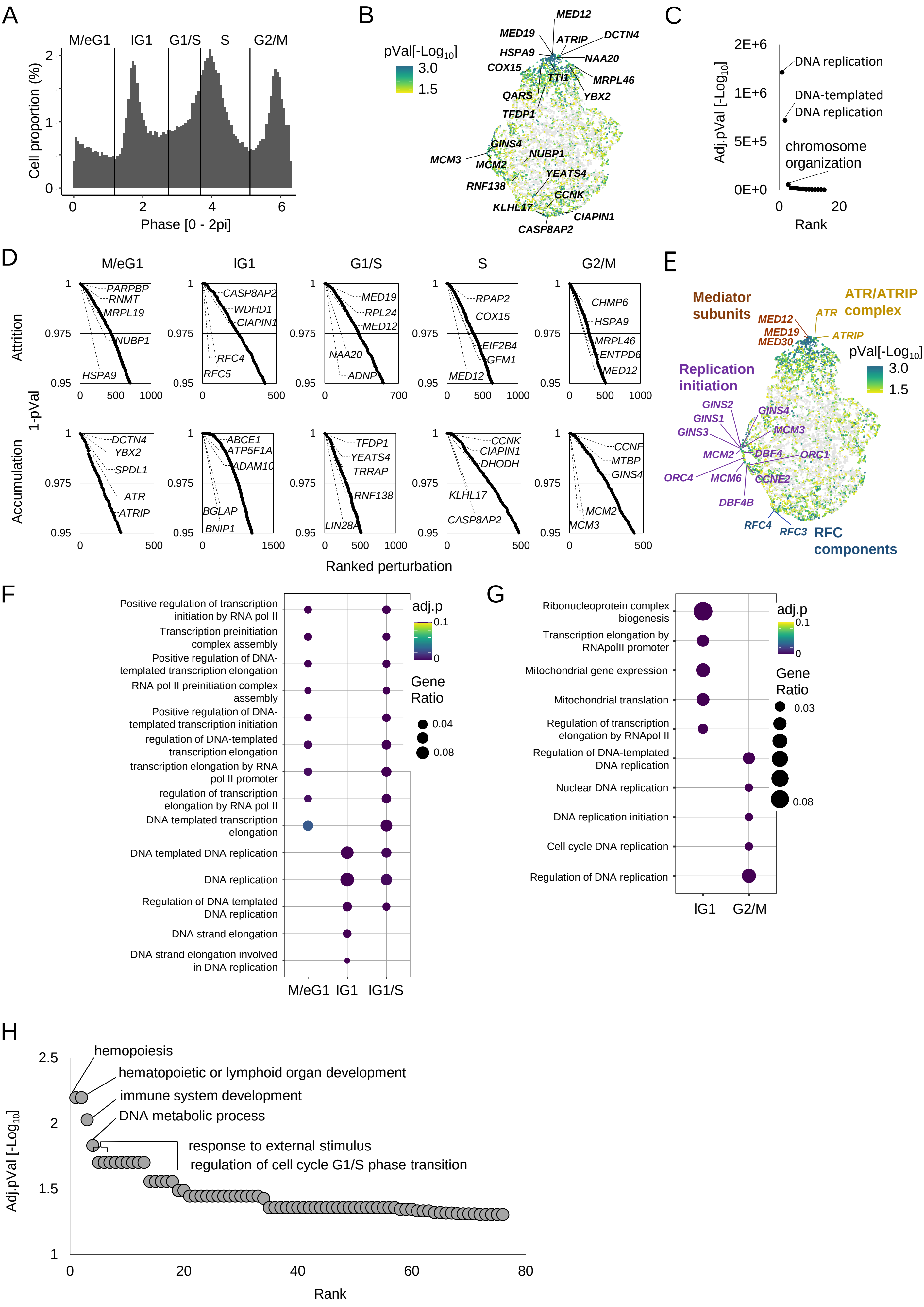
A
M/eG1
IG1
G1/S
S
G2/M
Cell proportion (%)
Phase [0 - 2pi]
B
pVal[-Log10]
3.0
1.5
MED12
MED19
ATRIP
DCTN4
HSPA9
NAA20
COX15
TTI1
MRPL46
YBX2
QARS
TFDP1
GINS4
MCM3
MCM2
NUBP1
RNF138
YEATS4
CCNK
KLHL17
CIAPIN1
CASP8AP2
C
Adj.pVal [-Log10]
2E+6
1E+6
5E+5
0E+0
DNA replication
DNA-templated DNA replication
chromosome organization
Rank
D
Attrition
Accumulation
1-pVal
Ranked perturbation
PARPBP
RNMT
MRPL19
NUBP1
HSPA9
CASP8AP2
WDHD1
CIAPIN1
RFC4
RFC5
MED19
RPL24
MED12
NAA20
ADNP
RPAP2
COX15
EIF2B4
GFM1
MED12
CHMP6
HSPA9
MRPL46
ENTPD6
MED12
DCTN4
YBX2
SPDL1
ATR
ATRIP
ABCE1
ATP5F1A
ADAM10
BGLAP
BNIP1
TFDP1
YEATS4
TRRAP
RNF138
LIN28A
CCNK
CIAPIN1
DHODH
KLHL17
CASP8AP2
CCNF
MTBP
GINS4
MCM2
MCM3
E
Mediator subunits
ATR/ATRIP complex
MED12
MED19
MED30
ATR
ATRIP
pVal[-Log10]
3.0
1.5
Replication initiation
GINS2
GINS1
GINS3
GINS4
MCM3
MCM2
DBF4
ORC1
ORC4
MCM6
CCNE2
DBF4B
RFC4
RFC3
RFC components
F
Positive regulation of transcription initiation by RNA pol II
Transcription preinitiation complex assembly
Positive regulation of DNA-templated transcription elongation
RNA pol II preinitiation complex assembly
Positive regulation of DNA-templated transcription initiation
regulation of DNA-templated transcription elongation
transcription elongation by RNA pol II promoter
regulation of transcription elongation by RNA pol II
DNA templated transcription elongation
DNA templated DNA replication
DNA replication
Regulation of DNA templated DNA replication
DNA strand elongation
DNA strand elongation involved in DNA replication
M/eG1
IG1
IG1/S
adj.p
0.1
0
Gene Ratio
0.04
0.08
G
Ribonucleoprotein complex biogenesis
Transcription elongation by RNApolII promoter
Mitochondrial gene expression
Mitochondrial translation
Regulation of transcription elongation by RNApol II
Regulation of DNA-templated DNA replication
Nuclear DNA replication
DNA replication initiation
Cell cycle DNA replication
Regulation of DNA replication
IG1
G2/M
adj.p
0.1
0
Gene Ratio
0.03
0.08
H
Adj.pVal [-Log10]
hemopoiesis
hematopoietic or lymphoid organ development
immune system development
DNA metabolic process
response to external stimulus
regulation of cell cycle G1/S phase transition
Rank

Figure S3. Perturb-seq-derived cell cycle imbalances, related to Figure 2

(A) Cell cycle phase distribution of K562 transduced with non-targeting guide RNAs[4] estimated using *VeloCycle*[3]. Phases were further split into five bins (M/eG1, lG1, G1/S, S, G2/M).
(B) UMAP projection of cell cycle imbalances calculated from a K562 Perturb-seq[4] with *VeloCycle*[3]. Each dot corresponds to one perturbation and is colored by the statistical significance of a test based on a multinomial with parameters derived from a binned, unperturbed cell cycle phase distribution (Fig. S1A). Non-significant imbalances ($p>0.05$) are shown in grey. The three genes leading to the most severe imbalances (either accumulation or attrition, smallest p-values) in each phase are highlighted.
(C) Enrichment of imbalance-inducing ($p < 0.05$) perturbation targets across GOBP terms, ranked by statistical significance (Benjamini-Hochberg adj. p-values, hypergeometric test)
(D) Perturbations inducing cell attritions (top) or accumulations (bottom) ($p < 0.05$) in the bins shown in (A).
(E) Same UMAP as in (Fig. S2B), highlighting perturbations inducing imbalances and targeting components of the same functional complexes or pathways.
(F) Enrichment of perturbations leading to attritions or accumulations (G) across GOBP terms.
(H) Enrichment of DBPs matching the following three criteria: (1) causing cell cycle imbalance when depleted, (2) enriched at the promoters of rhythmic genes, and (3) encoded by significantly rhythmic genes across GOBP terms (hypergeometric test).

A

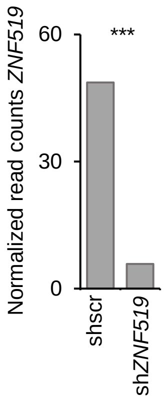

B

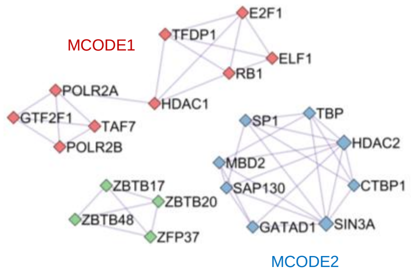

MCODE-1

Code	Description	pVal
R-HSA-3700989	Transcriptional Regulation by TP53	-11.9
M279 PID	RB1 PATHWAY	-11.3
GO:0006351	DNA-templated transcription	-10.7

C

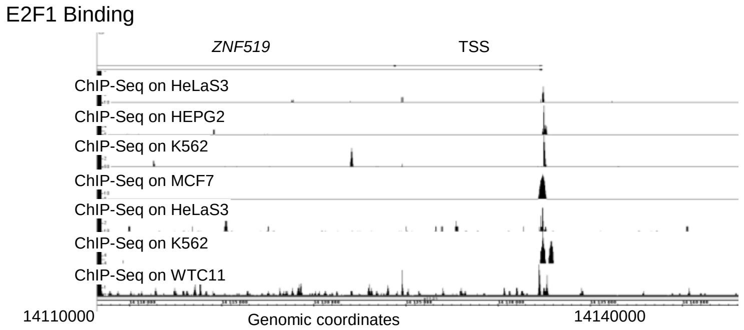

D

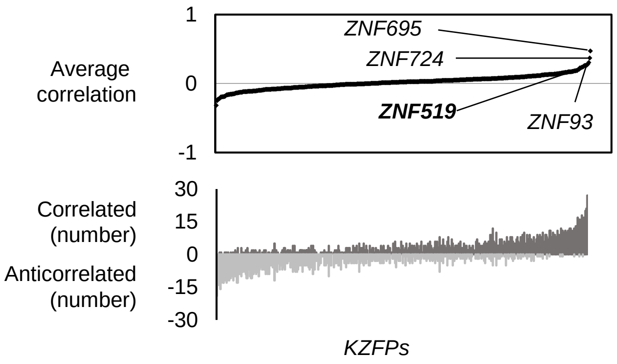

Figure S4. *ZNF519* promoter activity correlates with cell proliferation, related to Figure 3

(A) ZNF519 expression (RNA-seq) in shZNF519 versus shSCR K562 at day 4 post-transduction. Bars represent mean normalized RNA counts. n = 3, *** denotes a p-value < 0.005, moderated t-test.
(B) Protein-Protein Interaction Network built using DBPs bound to the promoter region of *ZNF519* (GeneHancer Identifier: GH18J014131, TSS distance = +0.1 Kb) and analysed with the mature complex identification algorithm, which allows for the identification and annotation of functional protein complexes. The table reports GO terms associated with the subnetwork MCODE1.
(C) IGB screenshot showing the *ZNF519* TSS. E2F1 binding measurements conducted in various cell lines are depicted in black[5].
(D) Top: Median Spearman's rank correlation of KZFP expression with 168 cell cycle and proliferation markers across 33 TCGA cancer subtypes. Bottom: Number of cancer subtypes in which KZFP expression is correlated (black) or anticorrelated (grey) with the proliferation signature (pVal <0.05).

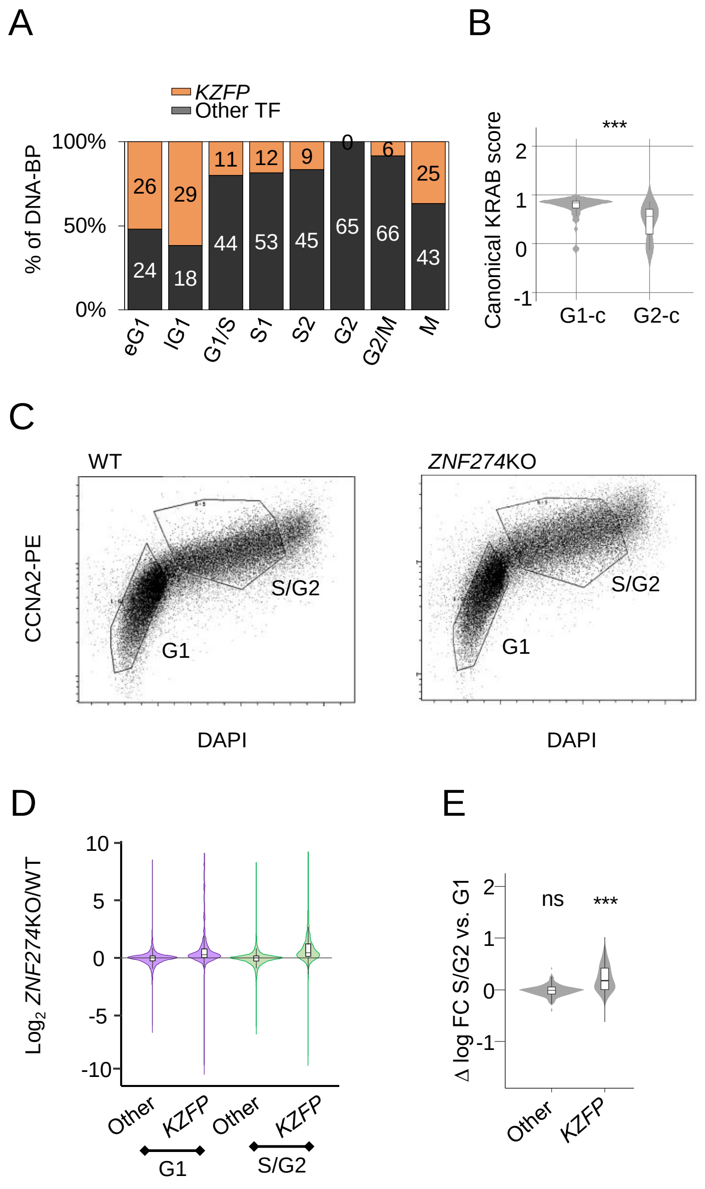

A
B
KZFP
Other TF
100%
50%
0%
% of DNA-BP
26
29
11
12
9
0
6
25
24
18
44
53
45
65
66
43
eG1
lG1
G1/S
S1
S2
G2
G2/M
M
Canonical KRAB score
***
2
1
0
-1
G1-c
G2-c
C
WT
ZNF274KO
CCNA2-PE
S/G2
G1
DAPI
D
E
10
5
0
-5
-10
Log2 ZNF274KO/WT
Other
KZFP
G1
S/G2
Δ log FC S/G2 vs. G1
ns
***

Figure S5. ZNF274 knockout specifically alters the cell cycle expression of *KZFPs*, related to Figure 4

(A) Proportion of rhythmic KZFPs and other TFs peaking in the different cell cycle phases.
(B) Prediction of the silencing strength (1 means optimal silencing) exerted by KRAB domains of G1-c and G2-c rhythmic KZFPs.
(C) *ZNF274* KO and wild-type 293T stained with anti-CCNA2 antibody and DAPI. G1 and S/G2 sorting gates are depicted in black.
(D) Log2 fold change expression of rhythmic KZFPs and other genes in *ZNF274* KO versus wild-type 293T in FACS-sorted G1 or S/G2 cells.
(E) Differential S/G2 versus G1 log2 fold change expression of rhythmic KZFPs and other genes in *ZNF274* KO versus wild-type 293T. "***" denotes a p-value < 0.005, Wilcoxon's test with $\mu = 0$.

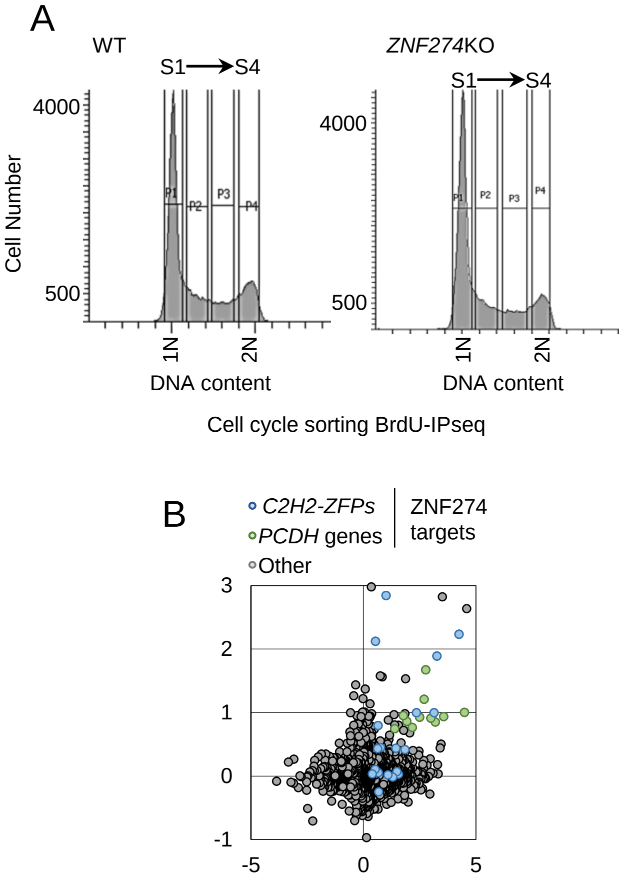
A
WT
S1 S4
4000
500
Cell Number
1N
2N
DNA content
ZNF274KO
S1 S4
4000
500
1N
2N
DNA content
Cell cycle sorting BrdU-IPseq
B
C2H2-ZFPs
PCDH genes
Other
ZNF274 targets
3
2
1
0
-1
-5
0
5

Figure S6. ZNF274 orchestrates late replication timing at non-*KZFP* target genes, related to Figure 5

(A) Cell cycle distribution of *ZNF274* KO and WT 293T stained with propidium iodide (PI). Sorting gates are depicting and annotated in black.

(B) Log2 fold change expression and differential RT index of C2H2 ZFP (blue) and PCDH genes (green) targeted by ZNF274, and other genes (grey) in *ZNF274* KO versus WT 293T.

Supplemental References

1. Cornelissen, G. (2014). Cosinor-based rhythmometry. Theor. Biol. Med. Model. *11*, 16. https://doi.org/10.1186/1742-4682-11-16.

2. Coudray, A., Forey, R., Haro, B.B., Martins, F., Carlevaro-Fita, J., Sheppard, S., Offner, S.E., La Manno, G., Obozinski, G., and Trono, D. (2023). Deconvolution of ex-vivo drug screening data and bulk tissue expression predicts the abundance and viability of cancer cell subpopulations. Preprint, https://doi.org/10.1101/2023.05.10.540140 https://doi.org/10.1101/2023.05.10.540140.

3. Lederer, A.R., Leonardi, M., Talamanca, L., Bobrovskiy, D.M., Herrera, A., Droin, C., Khven, I., Carvalho, H.J.F., Valente, A., Dominguez Mantes, A., et al. (2024). Statistical inference with a manifold-constrained RNA velocity model uncovers cell cycle speed modulations. Nat. Methods. https://doi.org/10.1038/s41592-024-02471-8.

4. Replogle, J.M., Saunders, R.A., Pogson, A.N., Hussmann, J.A., Lenail, A., Guna, A., Mascibroda, L., Wagner, E.J., Adelman, K., Lithwick-Yanai, G., et al. (2022). Mapping information-rich genotype-phenotype landscapes with genome-scale Perturb-seq. Cell *185*, 2559-2575.e28. https://doi.org/10.1016/j.cell.2022.05.013.

5. The ENCODE Project Consortium (2012). An integrated encyclopedia of DNA elements in the human genome. Nature *489*, 57–74. https://doi.org/10.1038/nature11247.
